# Merkel cell carcinoma: Lessons from a review of 15 year outcomes of the multidisciplinary management at a UK tertiary referral centre

**DOI:** 10.1016/j.jpra.2026.10.003

**Published:** 2026-10-23

**Authors:** Anna Y Allan, Khushboo Sinha, Hawys Lloyd-Hughes, Alastair MacKenzie Ross, Irene De Francesco, Bjorn Thomas, Zena Willsmore, Dhruba Dasgupta, Sean Whittaker, Eduardo Calonje, Mark Harries, Yin Wu, Jenny LC Geh

**Affiliations:** a Plastic Surgery Department & Skin Tumour Unit, Guy’s and St Thomas’ Hospital NHS Foundation Trust. London, UK; b St John’s Dermatology & Skin Tumour Unit, Guy’s and St Thomas’ Hospital NHS Foundation Trust. London, UK; c Oncology & Skin Tumour Unit, Guy’s and St Thomas’ Hospital NHS Foundation Trust. London, UK; d Radiology & Skin Tumour Unit, Guy’s and St Thomas’ Hospital NHS Foundation Trust. London, UK; e Histopathology and Skin Tumour Unit, Guy’s and St Thomas’ Hospital NHS Foundation Trust. London, UK; f Plastic Surgery Department, North West Anglia NHS Foundation Trust, UK

**Keywords:** Merkel cell carcinoma, MCC, Skin cancer, Neuroendocrine carcinoma, Surgical oncology, Skin oncology, Sentinel lymph node biopsy, Cancer management, Immunotherapy, Treatment outcomes, Multidisciplinary care, Team-working

## Abstract

**Introduction:**

Merkel cell carcinoma (MCC) is a rare, aggressive skin cancer with high mortality, low survival and propensity for recurrence and metastasis. Worldwide data on MCC patient outcomes are limited. What can we learn from our experience of a UK tertiary referral centre, over a 15 year period?

**Methods:**

Guy’s Cancer Centre registry was retrospectively reviewed for MCC patients diagnosed between 2008 and 2022. Electronic medical records were reviewed for demographics, tumour characteristics, treatment modalities and outcomes including all-cause mortality. We aimed to identify the incidence and outcomes from the multidisciplinary management of this rare disease.

**Results:**

81 cases of MCC were included (55% male, mean age at presentation 71.4 years, range 40–93 years, SD 11.2). Mean follow-up was 4.1 years. 40% presented with metastatic disease, 42% of these had regional lymph node metastases. 15 patients underwent sentinel lymph node biopsy (SLNB), 40% of these were positive. SLNB provided accurate staging and guided adjuvant treatment but did not appear to confer a survival benefit. Adjuvant treatments of radiotherapy and systemic therapy have evolved to be added in over the years.

**Conclusions:**

This single-centre study examines long-term MCC outcomes in a UK population that is comparable to published data. Our data suggests SLNB positivity may confer prognostic value. We suggest rapid and earlier treatment is best. Hence working these patients up in a specialist skin (Melanoma) MDM makes sense given the options of adjuvant radiotherapy and systemic therapies, where adverse events can be managed. Given the paucity of data worldwide, we hope our experience will support clinicians in counselling patients on treatment options. Survival data alone may not represent the optimum measure of a good outcome. Non-clinical factors, such as social support and transport issues may influence the outcomes more than final treatment given. Patients’ and their care-givers’ partnerships should be encouraged in the decision-making process. Treatment accepted may not reflect pathways of guidance proffered due to the changing landscape of options and individual patient choice.

## Graphical abstract

**Figure:**
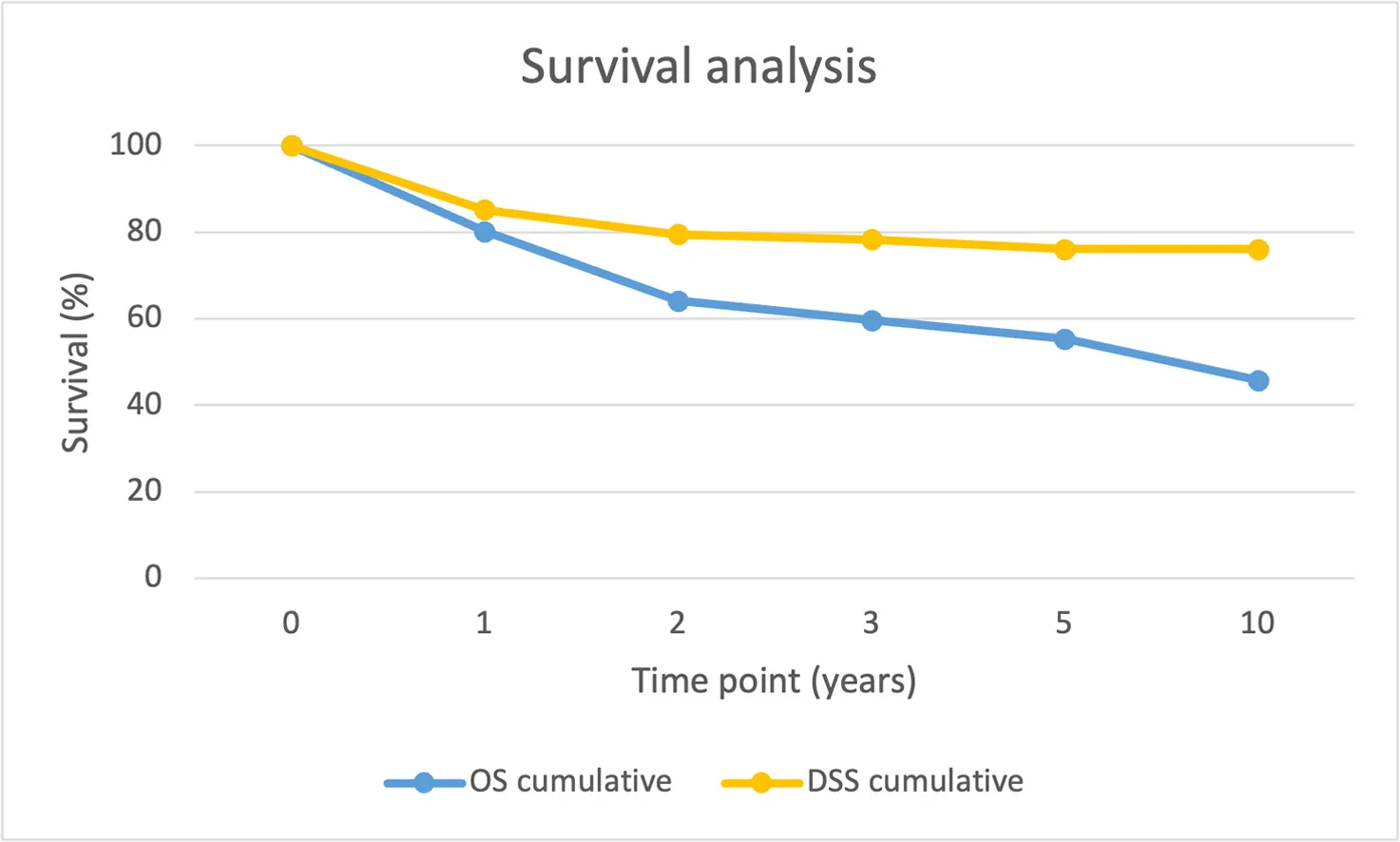

## Introduction

Merkel cell carcinoma (MCC) is a rare, highly aggressive, primary cutaneous neuroendocrine malignancy, first described by Toker as trabecular carcinoma of the skin.^1^ It carries high mortality and has a propensity for recurrence and metastatic potential. Although incidence remains low at 0.65 per 100,000 person-years, cases are rising in the United Kingdom.^2^^,^^3^ Risk factors include advancing age, ultraviolet light exposure.^4^ Merkel cell polyomavirus (MCPyV) infection^5^^,^^6^ and those with immune-compromise, particularly T-cell deficiency.^7^ MCPyV prevalence is higher in the northern hemisphere compared to Australian tumours.^8^ MCC has a predilection for the head and neck.^3^ A decreased incidence of MCC is noted in the younger (<50years) age group in the United States by Alves and colleagues.^9^ They propose that protection from UV exposure could be main reason for observing this pattern. Its rarity poses challenges when developing effective prevention strategies and targeted therapies, with limited worldwide outcome data. Current staging uses the American Joint Committee on Cancer/Union for International Cancer Control 8th Edition, based on 9387 cases from the National Cancer Database.^10^ For stages I-III, surgery with 1–2 cm margins is the recommended primary treatment based on international guidelines, with radiotherapy reserved for post-operative high-risk cases, close margins or inoperable patients.^11^^,^^12^ Recent immunotherapy advances have transformed MCC treatment with checkpoint inhibitors newly incorporated for advanced disease.^10^^,^^13^

We describe a single UK tertiary referral centre experience of MCC over a 15 year period, with evolving provision of sentinel node biopsy, radiotherapy and immunotherapy. This study aims to contribute data to a rare condition that lacks outcome data. We are a primary, secondary and tertiary referral centre, with a multi-speciality meeting (MDM), comprising dermatology, plastic surgery, medical & clinical oncology, histopathology, radiology and specialist nurses. The MDM runs before a joint clinic of dermatology, plastics and oncology. There is access, with close proximity to Peri-operative Older Persons’ (POPS) and other speciality clinics such as cytology, Mohs micrographic surgery and other teams (ophthalmology, head and neck and urology) for rapid input and opinions. Best interest meetings and access to our research teams can be arranged in the same building over subsequent visits if needed. Our MDM team is called the Skin Tumour Unit at St John's Dermatology and forms part of the Guy's Cancer Centre set up for cancer care and research.

## Methods

This retrospective single-centre review was approved by the Guy’s and St Thomas’ audit department. The Guy’s Cancer Centre registry was screened for MCC patients diagnosed between 2008–2022. Electronic medical records were reviewed for patient demographics, tumour characteristics, imaging, treatment modalities and outcomes including all-cause mortality.

This study was conducted in accordance with Guy’s and St Thomas’ Hospital data protection rules and the Declaration of Helsinki (October 2024).

Metastatic disease was classified as: satellite lesions (local), lymph node basins that would typically drain the primary tumour site (regional) and additional sites (distant metastases).

When defining co-morbidities, cerebrovascular accidents and dementia were classified as neurological conditions even though it is noted that the underlying cause is vascular disease. Systemic therapies were recorded as were their outcomes.

## Results

83 patients with MCC were identified; 2 patients were excluded as they had treatment at another institution, leaving 81 cases for analysis. This gave us an incidence of 0.38 per 100,000, slightly lower than that quoted in the paper by Mistry.^2^ Mean follow up was 4.1 years (range 365–5459 days). Males comprised 56% of cases. Mean age at presentation was 71.9 years (range, 40–93, SD 10.8). All cases were confirmed histologically. Co-morbidities are described in Table 1. 38% (31/81) presented with metastatic disease; 42% (13/31) of these had regional lymph node metastases.

**Table 1:** Patient and tumour characteristics.

	Mean ± SD or no. (%)
**Age at diagnosis y**	71.9 ± 10.8
**Sex**	
Female	36 (44)
Male	45 (56)
**Stage at diagnosis**	
Stage 1	2
Stage 2	48
Stage 3	4
Stage 4	29
**Site of primary tumour**	
Head and neck	30 (37)
Upper limb	19 (23)
Lower limb	18 (22)
Torso	5 (6)
Unknown primary	9 (11)
**Metastatic disease at presentation**	
Y	31 (38)
N	50 (62)
**Sites of metastatic disease**	
Local	4 (13)
Regional	13 (42)
Distant	5 (16)
Unknown primary	9 (29)
**Co-morbidities**	
Cardiac	48 (59)
Respiratory	17 (21)
Gastrointestinal	8 (10)
Neurological	25 (31)
Diabetes	18 (22)
Immunocompromised	13 (16)
Previous other malignancy	34 (42)
Average number of co-morbidities per patient at time of diagnosis	3.6 ± 2.3

### Primary treatment

First-line treatment comprised surgery (71%, 57/81), radiotherapy (14%, 11/81) and systemic therapy (6%, 5/81), compared with Mistry’s 80.7% of patients, who underwent surgical excision, 43.5% radiotherapy and 9.2% systemic therapy. Details of patient management are detailed in Fig. 1, Fig. 2, Fig. 3. 7% (6/81) declined surgery and 2% (2/81) died from MCC before treatment initiation. Surgical details are in Table 2. 67% (29/43) had 20 mm margins, 12% (5/43) had 30 mm margins and 21% (9/43) had ≤15 mm margins due to anatomical constraints. At wide local excision, 79% (34/43) patients had adequate surgical margins, 12% (5/43) had close margins (<1 mm) and 9% (4/43) had incomplete deep margins.

**Fig. 1:**
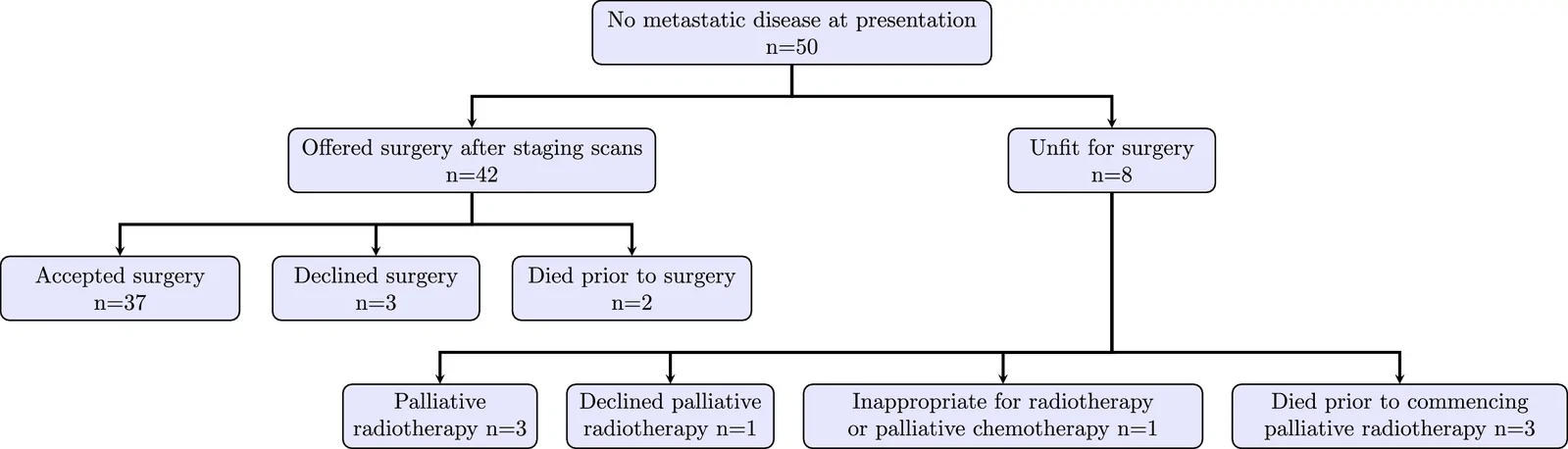
Patient management overview without metastatic disease at presentation.

**Fig. 2:**
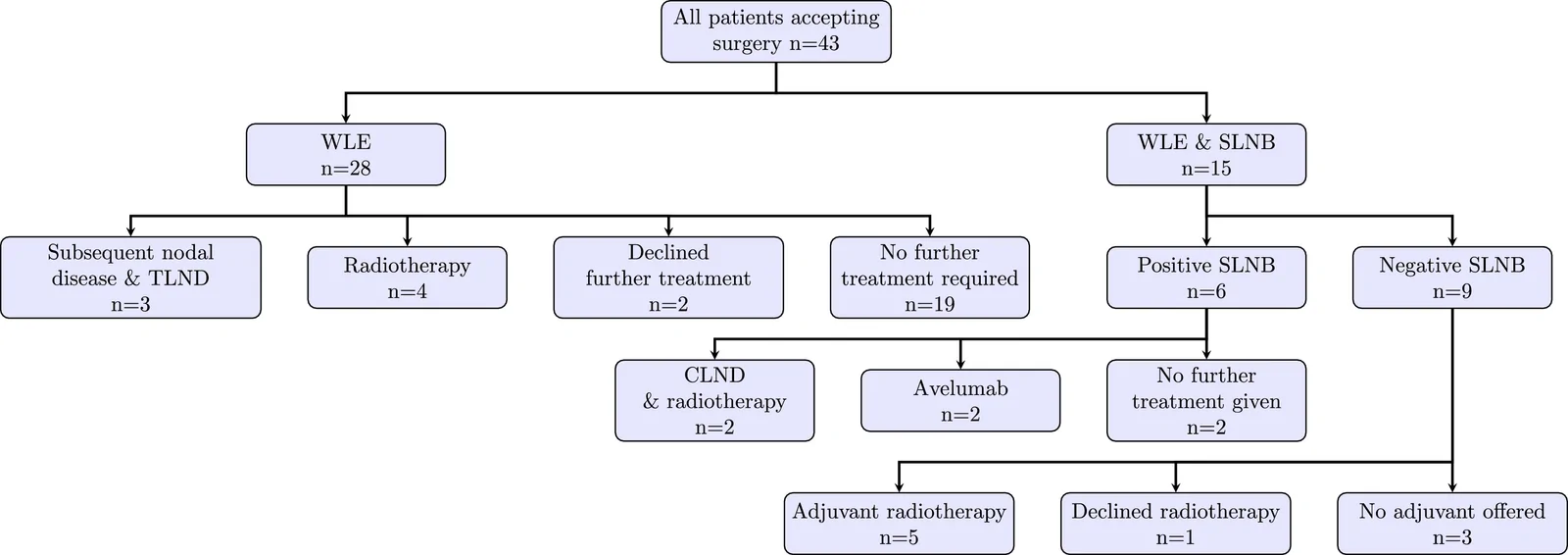
Patient management for non-metastatic disease undergoing surgery. WLE: Wide Local Excision; SLNB: Sentinel Lymph Node Biopsy; TLND: Therapeutic for.

**Fig. 3:**
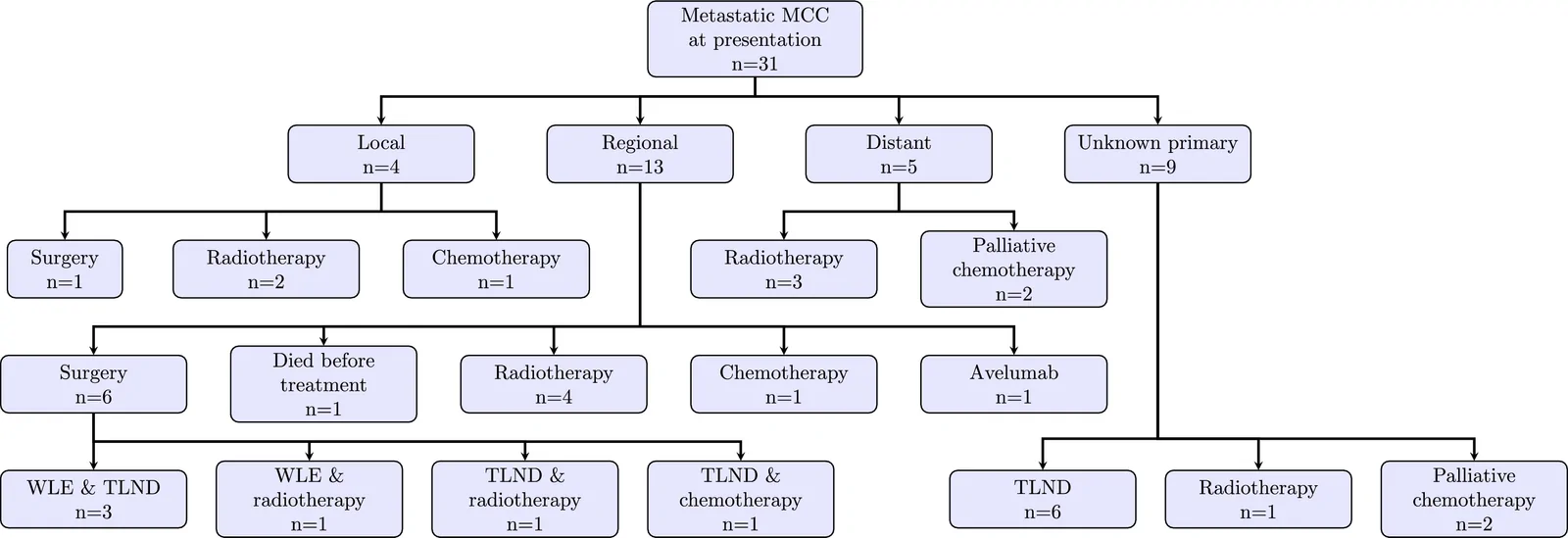
Patient management overview with metastatic disease at presentation. WLE: Wide Local Excision; TLND: Therapeutic for Lymph Node Disease Dissection.

**Table 2:** Treatment summary.

**Treatment**	**Number (%)**	**Details**
**Surgical Treatment offered**	59	
WLE	43	
SLNB	19	
Open biopsy of lymph node	3	
Lymphadenectomy	6	
**Surgical WLE margins**	43 patients	
5mm	1 (2)	Nasal vestibule
6mm	1 (2)	Upper eyelid
10mm	5 (12)	Elbow, finger, foot, forearm (patient declined 20 mm)
15mm	2 (5)	Nasal bridge, elbow (patient unfit for GA to facilitate 20 mm)
20mm	29 (67)	
30mm	5 (12)	Scalp x3, Thigh x2
**Reconstruction**	43 patients	
Direct closure	18 (42)	
Artificial dermal matrix alone	5 (12)	
Artificial dermal matrix + SSG	4 (9)	
SSG	5 (12)	
FTSG	5 (12)	
Local flap	5 (12)	
Free flap	1 (2)	
**Adjuvant therapy**	28 patients	
Radiotherapy	26 (93)	
Chemotherapy	2 (7)	
**Treatment for metastatic disease**	12 patients	
Palliative chemotherapy	6 (50)	
Systemic therapy (Avelumab)	6 (50)	One patient offered but declined (total offered Avelumab = 7)

Overall five-year all-cause mortality was 59% (41/70); disease-specific death (DSD) accounted for 50% (20/70). Of the remaining patients (41%, 29/70), 97% (28/29) had no recurrence at five years. Overall and disease-free survival is represented in Graph 1. We have summarised an overview of patient management in Fig. 1, Fig. 2, Fig. 3.

### Sentinel lymph node biopsy (SLNB)

40% (6/15) of sentinel lymph node biopsies (SLNB) were positive. In those with positive SLNB (6/15), two (33%, 2/6) received adjuvant radiotherapy, two underwent completion lymph node dissection and two received Avelumab. A summary of patients who accepted Avelumab and their outcomes is in Appendix 1. Five-year all-cause mortality was 33% (2/6); disease-specific death accounted for 50% (1/2). None of the remaining patients (67%, 4/6), had recurrence at five years.

In those with a negative SLNB (9/15), five (56%, 5/9) underwent adjuvant radiotherapy to the primary site due to close margins. Five-year all-cause mortality was 11% (1/9). This patient died of metastatic MCC. 78%, (7/9) did not have recurrence at five years and one patient (11%, 1/9) reached two years follow up with no recurrence.

## Discussion

In the UK, MCC incidence is 0.65 per 100,000 person-years, translating to approximately 30–35 patients per specialist unit every five years.^2^ Our median age at presentation is similar to other published sources.^2^ Our unit manages a high volume of MCC patients.^14^, ^15^, ^16^ Assessment and management remain debated due to MCC’s rarity. Our findings align with similar studies: MCC predominantly affects elderly individuals, commonly involves the head and neck and carries a poor prognosis for both recurrence and in the presence of metastatic disease. SLNB is offered, but often declined. SLNB positivity is high,(40%), making the upstaging of MCC important for discussions on adjuvant radiotherapy or systemic therapy.

Our management strategies have evolved over the study period (2008–2022), transitioning from surgery ± radiotherapy to incorporating checkpoint inhibitors, for metastatic disease. Our unit was the first to publish a series of Robotic lymph node dissection for skin cancer pelvic metastases.^17^ We now offer Avelumab for systemic disease as either first line or after chemotherapy. Chemotherapy with Carboplatin and Etoposide for metastatic disease can be first line or after Avelumab. This evolution complicates patient counselling, requiring clinicians to convey overall prognosis, treatment options and impact on quality of life through informed, shared decision-making.^18^ The use of systemic therapies means that support for acute oncological side effects must be supported in a manageable way for these patients. In our elderly cohort, the counselling process itself materially influenced treatment selection, with some patients opting against more aggressive surgical intervention, perhaps due to age-related considerations. All cases with significant co-morbidities and older age are routinely offered an older persons assessment in a separate MDM. We are fortunate to work closely with a well-established peri‑operative older persons’ (POPS) pathway team.^19^^,^^20^ This is a legitimate outcome of shared decision-making in this population and should not be considered a failure of care.

Our data adheres to NCCN guidelines regarding excision margins.^11^ Routine inclusion of the deep fascia achieved 80–100% complete excision rates. Recent studies report no differences in survival between positive versus negative margins with post-operative radiotherapy.^21^^,^^22^ Whilst our sample size is insufficient to corroborate these findings within a UK population, they suggest a potential shift toward more conservative surgical approaches. Mohs micrographic surgery, whilst described in the literature, is not usually offered in our unit for MCC. Wide local excision, with the offer of SLNB and subsequent post-operative radiotherapy, achieves comparable oncological control and remains the standard of care in our unit. Modern protocols (such as local or regional anaesthesia and improved recovery) have reduced morbidity, making surgery feasible even for those with significant co-morbidities. There is little dispute that surgery alone remains the one of the most cost-effective means of controlling all types of skin cancer and potentially achieving cure, particularly for early-stage disease. However, MCC is unusual in being very radiosensitive.

Radiotherapy remains an important adjunct therapy with well-supported efficacy^23^, ^24^, ^25^, ^26^, ^27^ though logistical challenges, such as repeat visits, frequently deter elderly patients. Australian practice favours definitive radiotherapy for early-stage MCC,^28^ contrasting with UK approaches. The RATIONAL trial aimed to look at whether surgery or radiotherapy is superior for initial loco-regional control.^29^ It had to close because it was not possible to randomise patients, and therefore both modalities remain standard pillars of MCC management. The GoTHAM trial (NCT04261855) aims to clarify the added value of radiotherapy to checkpoint inhibitors in first-line metastatic MCC management.

Checkpoint inhibitors have transformed metastatic MCC treatment. Avelumab (Bavencio) is an approved PD-L1 immune checkpoint inhibitor for metastatic MCC. It is a fully human monoclonal antibody that blocks the interaction between PD-L1 on tumour cells and PD-1 on T-cells, thereby inhibiting immunosuppression in the tumour microenvironment and reducing tumour growth. It is administered by infusion every two weeks. Standard side-effect profile and monitoring mandates that it is given by a team who have the full support of an acute oncology service. In 2017, Avelumab became the first approved treatment for metastatic MCC based on the JAVELIN Merkel 200 trial^12^^,^^30^ with five-year data confirming survival benefit.^31^ Pembrolizumab (PD-1 inhibitor) is also approved in the USA based on the KEYNOTE-017 trial.^32^ Despite higher progressive diseases rates, re-treatment shows promise.^33^ Whilst potential side effects and requirement for acute oncology support present challenges, future directions including biological combination therapies hold promise for improving treatment efficacy while reducing toxicity. The role of adjuvant ICI therapy remains undetermined; interim ADMEC—O trial data suggest adjuvant Nivolumab, a PD-1 inhibitor, may reduce recurrence risk, although overall survival data remain immature.^34^

Our five-year all-cause mortality is 59%, with disease-specific mortality comprising approximately half. These statistics raise critical questions regarding optimal management strategies. We advocate routine inclusion of MCC cases in specialist multidisciplinary meetings to ensure comprehensive treatment planning with medical and clinical oncology input. Given the similarities in support around SLNB and immunotherapy treatments, specialist Melanoma MDMs may be more appropriate than non-melanoma MDMs in the UK.

## Key principles guiding our practice

• Early recognition and diagnosis - avoid delays in referral
• Complete excision at initial presentation as incision/partial biopsies can create delays in pathology review
• Accurate staging (via SLNB/imaging)
• Multidisciplinary discussion of all treatment options
• Joint patient and care-giver decision-making
• Manage in the specialist Melanoma MDM clinic (allows for acute oncology support for systemic therapies)

## Limitations

This single-institution retrospective review has inherent limitations. Small individual cohorts limit conclusions. Additionally, the 15-year study period encompasses significant advances, including the introduction of immunotherapy.

A note on the question of multicentre collaboration is warranted. While pooling data from multiple centres would increase sample size, it would also introduce demographic heterogeneity. The south-eastern, northern and south-western sectors of London are known to differ meaningfully in their population profiles, and combining cohorts from these areas risks conflating distinct demographic contexts. This must be carefully considered in the design of any future collaborative study, with robust methodology to account for such variation. The evolving management of MCC could be influenced by the onset of next generation sequencing (NHS Cancer Plan), in particular with systemic therapies and the link of the polyomavirus as a causative agent.^6^, ^7^, ^8^^,^^35^

A national MCC registry with standardised reporting and minimum dataset, including RCPath histopathological parameters, is needed. There is currently no validated, standardised outcome measure for satisfaction or wellbeing in the elderly MCC population. For this group, quality of life is likely to represent a more meaningful metric than survival duration alone; a patient’s preference for less burdensome treatment, even at some oncological cost, is a legitimate and rational choice. Existing tools such as the Cancer Quality of Life Survey provide a starting point, but instruments specifically validated for older patients with aggressive cutaneous malignancy are lacking and represent an important area for future development. Integrating patient-reported outcomes, mental health assessments, and psychosocial metrics into any national registry would promote holistic care and address currently unmet needs.

Accurate prognostication supports appropriate patient counselling and decision-making. We agree with Andrew et al’s use of a multivariate FG prediction model.^36^ Personalised survival predictions leveraging machine-learning prognostic tools such as DeepMerkel^37^ may support challenging patient and MDT discussions. For this older population, quality of life may be a more valuable metric than the simplistic measure of survival.

## Conclusion

This study provides UK-specific data to inform shared decision-making for newly diagnosed MCC patients, enhancing understanding of treatment rationale and expected survival. Delivering the full range of treatments within a single specialist centre is likely to support better outcomes through integrated expertise and streamlined pathways. Development of a national minimum dataset will be instrumental in refining treatment strategies and enhancing quality of life outcomes. We emphasise that early, complete surgical excision offers the best opportunity for improved outcomes in terms of both survival and patient wellbeing. These efforts represent a necessary step towards addressing the numerous unanswered questions surrounding this challenging malignancy.

## Ethical approval

Not required.

## Funding

None.

## Declaration of competing interest

None declared.
