# Supplementary material for: Merkel cell carcinoma: Lessons from a review of 15 year outcomes of the multidisciplinary management at a UK tertiary referral centre
Source: JPRAS Open. 2026 Oct 23;53:119–27. doi: 10.1016/j.jpra.2026.10.003 (PMC13762899; doi:10.1016/j.jpra.2026.10.003)
Supplement: Supplementary file 1 [file mmc1.docx]

Appendix 1: Summary of treatment and outcomes for patients who completed Avelumab treatment

| Patient | Primary tumour | Metastatic disease on presentation | Treatment offered at presentation | Progression | Outcome |
| --- | --- | --- | --- | --- | --- |
| 1 | Lower limb | N | WLE + SLNB  Positive SLNB. Post-operative poor healing so radiotherapy to nodal basin only | Metastatic disease noted to H&N and lower limb. Avelumab offered. | Progression despite Avelumab. Good response to radiotherapy. Progression despite chemotherapy. Referred for palliative care |
| 2 | Cheek | N | WLE + SLNB. MCC extended to deep margin of WLE, positive SLNB | Rapid tumour growth noted so Avelumab offered in view of residual disease and nodal basin involvement | No clinical recurrence. Death secondary to new lung adenocarcinoma |
| 3 | Eyelid | N | WLE alone | Recurrence noted. Therapeutic LN dissection and parotidectomy. Post-operative chemoradiotherapy completed. 3 years later adrenal mets noted and resected. Commenced on Avelumab in view of distant metastatic disease | No clinical recurrence since completion of Avelumab |
| 4 | Eyelid | Y | Patient declined orbital extenteration. Patient accepted surgical tumour debulk only | Patient declined surgical treatment and radiotherapy; Avelumab offered | No clinical recurrence since completion of Avelumab |
| 5 | Eyelid | Y | Presented with locally advanced disease. | Declined surgery as too comorbid. Avelumab offered 4 cycles completed. Delayed response | No clinical recurrence. RIP secondary to respiratory disease |
| 6 | Torso | Y | PET-CT confirmed extensive nodal metastasis right groin with retro-peritoneal and infra-renal nodes. Offered radiotherapy to primary and Avelumab | Good response to radiotherapy but developed T1DM as toxicity to Avelumab | No clinical recurrence since completion of Avelumab |
